# Multimodal hybrid imaging agents for sentinel node mapping as a means to (re)connect nuclear medicine to advances made in robot-assisted surgery

**DOI:** 10.1007/s00259-015-3292-2

**Published:** 2016-01-15

**Authors:** Gijs H. KleinJan, Nynke S. van den Berg, Jeroen de Jong, Esther M. Wit, Helene Thygessen, Erik Vegt, Henk G. van der Poel, Fijs W. B. van Leeuwen

**Affiliations:** Interventional Molecular Imaging Laboratory, Department of Radiology, Leiden University Medical Hospital, Albinusdreef 2, 2300RC Leiden, The Netherlands; Department of Nuclear Medicine, The Netherlands Cancer Institute – Antoni van Leeuwenhoek Hospital, Plesmanlaan 121, 1066CX Amsterdam, The Netherlands; Department of Urology, The Netherlands Cancer Institute – Antoni van Leeuwenhoek Hospital, Plesmanlaan 121, 1066CX Amsterdam, The Netherlands; Department of Pathology, The Netherlands Cancer Institute – Antoni van Leeuwenhoek Hospital, Plesmanlaan 121, 1066CX Amsterdam, The Netherlands; Department of Biostatistics, The Netherlands Cancer Institute – Antoni van Leeuwenhoek Hospital, Plesmanlaan 121, 1066CX Amsterdam, The Netherlands; Department of Head and Neck Surgery & Oncology, The Netherlands Cancer Institute – Antoni van Leeuwenhoek Hospital, Plesmanlaan 121, 1066CX Amsterdam, The Netherlands

**Keywords:** Sentinel node, SPECT/CT, Prostate cancer, Fluorescence-guided surgery, Robot-assisted surgery

## Abstract

**Purpose:**

Radical prostatectomy and complementary extended pelvic lymph node dissection (ePLND) of sentinel lymph nodes (SNs) and non-sentinel lymph nodes (LNs) at risk of containing metastases are increasingly being performed using high-tech robot-assisted approaches. Although this technological evolution has clear advantages, the physical nature of robotic systems limits the integrated use of routine radioguided surgery technologies. Hence, engineering effort in robotics are focused on the integration of fluorescence guidance technologies. Using the hybrid SN tracer indocyanine green-^99m^Tc-nanocolloid (radioactive and fluorescent), for the first time in combination with a robot-integrated laparoscope, we investigated whether the robot-assisted approach affects the accuracy of fluorescence detection of SNs identified preoperatively using nuclear medicine.

**Methods:**

The study included 55 patients (Briganti nomogram-based risk >5 % on LN metastases) scheduled for robot-assisted radical prostatectomy, SN biopsy and ePLND. Following indocyanine green-^99m^Tc-nanocolloid injection, preoperative nuclear imaging (lymphoscintigraphy and SPECT/CT) was used to locate the SN(s). The fluorescence laparoscope was used intraoperatively to identify the SN(s) with standard fluorescence settings (in 50 patients) and with customized settings (in 5 patients). The number and location of the SNs, the radioactive, fluorescence (both in vivo and ex vivo) and tumour status of the resected SNs/LNs, and postoperative complications were recorded and analysed.

**Results:**

Combined, preoperative lymphoscintigraphy and SPECT/CT imaging identified 212 SNs (median 4 per patient). Intraoperative fluorescence imaging using standard fluorescence settings visualized 80.4 % (148/184 SNs; 50 patients; ex vivo 97.8 %). This increased to 85.7 % (12/14 SNs; 5 patients; ex vivo 100 %) with customized fluorescence settings. SPECT/CT images provided guidance towards the residual SNs. Ex vivo all removed SNs were radioactive. SNs were tumour-positive in 25.4 % of patients (14/55; false-negative rate 7 %, 1/14 patients). In ten patients, the SN was the only tumour-positive LN. Surgical complications were minimal.

**Conclusion:**

Directly linking 3D preoperative nuclear imaging information on SNs to a robot-integrated fluorescence laparoscope improved the surgeon’s use of the technology and did not influence the sensitivity or morbidity of the procedure. To our surprise, however, the detection rates with the current fluorescence camera did not improve.

## Introduction

In complex anatomies, accurate preoperative mapping of sentinel nodes (SNs) using nuclear medicine (lymphoscintigraphy with or without SPECT/CT) and intraoperative radioguidance are vital for planning and performing nodal resection [1, 2]. Imaging in combination with the intraoperative use of gamma probes and/or portable gamma cameras has been shown to be valuable not only in patients with melanoma, penile, vulvar and breast cancer [3–5], but also during the sampling of pelvic SNs originating from prostate cancer [6–8]. With SPECT/CT it has become possible to accurately identify the anatomical location of SNs inside or outside the extended pelvic lymph node dissection (ePLND) template, and subsequently this information can be used for surgical planning.

In parallel with the technical evolution of nuclear medicine-based imaging, there has been a shift towards robot-assisted laparoscopic procedures in urology, and in particular for prostate cancer. Unfortunately, this shift has resulted in a mismatch between the two disciplines. Moreover, in robotic surgery, the urologist is no longer present alongside the patient, limiting the control he/she has on the use of imaging technologies without robot assistance such as the gamma probe [9]. At the same time, the robot arms physically restrict access for larger devices such as portable gamma cameras. On the positive side, new-generation laparoscopes can be equipped with an integrated fluorescence imaging option [10–12]. Hence, integration of this technology in the robotic workflow currently seems to be more straightforward. Fluorescence guidance towards SNs and non-sentinel lymph nodes (LNs) has been achieved in a robotic setting through the use of an additional fluorescence laparoscope and via a robot-integrated fluorescence laparoscope [9–11, 13]. Unfortunately, the fluorescent signal has very limited tissue penetration, meaning that more deeply lying SNs/LNs may be missed when using fluorescence imaging alone [14]. Even worse, extensive surgery in the quest for a fluorescent signal may lead to an increase in surgical complications.

In 2009, the hybrid tracer indocyanine green- ^99m^Tc-nanocolloid (ICG-^99m^Tc-nanocolloid) was clinically introduced for SN biopsy. This tracer was designed to extend the European standard in nuclear medicine-based SN identification, ^99m^Tc-nanocolloid, with intraoperative fluorescence guidance [10, 15–17]. With this development, the strengths of radioguided procedures (e.g. a high sensitivity and in-depth view of the SNs) are complemented by detailed real-time, but superficial, fluorescence guidance towards the preoperatively identified SNs. In combination with the introduction of an additional fluorescence laparoscope, the hybrid concept has provided a significant step forward in connecting preoperative lymphatic mapping and real-time intraoperative fluorescence-based SN identification [9]. However, the fact that the bedside assistant had to introduce and control the fluorescence laparoscope, rather than the operating urologist, was considered a limiting factor of this approach. We thus hypothesized that use of a robot-integrated fluorescence laparoscope could increase the level of control the operating urologist has, and could thus help increase the success rate in resecting preoperatively identified SNs via fluorescence guidance. To place these findings in perspective, the tumour-positive rate, sensitivity, false-negative rate and complication rate of SN biopsy using the this approach was evaluated and compared to SN biopsy in a historical cohort [9].

## Materials and methods

### Patients

Between January 2014 and July 2015, 50 patients with a Briganti nomogram-based risk of >5 % of LN metastases were included [18]. These patients were evaluated with the robot-integrated fluorescence laparoscope (standard settings). Between July 2015 and September 2015 another 5 patients were included for evaluation of the customized fluorescence imaging settings. All patients were scheduled for robot-assisted radical prostatectomy, ePLND and SN removal using the hybrid tracer, and provided written informed consent. The characteristics of the two groups of included patients are shown in Table 1.

**Table 1:** Patient characteristics

	Group		Total
	SN + ePLND	SN + ePLND, customized settings	
No. of patients	50	5	55
Age (years), median (IQR)	63 (58 – 68)	66 (62 – 68)	63 (59 – 68)
Preoperative PSA level (ng/mL), median (IQR)	7.75 (5.44 – 12.18)	7.9 (4.8 – 23.35)	7.9 (5.46 – 12)
Clinical T stage, *n* (%)			
1c	6 (12)	–	6 (11)
2a	5 (10)	–	5 (9)
2b	9 (18)	1 (20)	10 (18)
2c	14 (28)	1 (20)	15 (27)
3a	12 (24)	2 (40)	14 (25)
3b	4 (8)	1 (20)	5 (9)
Biopsy Gleason sum score, *n* (%)			
6	3 (6)	–	3 (5)
7	30 (60)	4 (80)	34 (62)
8	15 (30)	1 (20)	16 (29)
9	2 (4)	–	2 (4)
Briganti score, median (IQR)	28 (21 – 53)	54 (20 – 68)	28 (21 – 50)
Clinical N stage, *n* (%)			
N0	44 (88)	5 (100)	49 (89)
Nx	6 (12)	–	6 (11)
Pathological T stage, *n* (%)			
2a	–	1 (20)	1 (2)
2b	3 (6)	–	3 (5)
2c	37 (74)	2 (40)	39 (71)
3a	4 (8)	1 (20)	5 (9)
3b	4 (8)	1 (20)	5 (9)
4	1 (2)	–	1 (2)
4a	1 (2)	–	1 (2)
Pathological Gleason sum score, *n* (%)			
6	6 (12)	1 (20)	7 (13)
7	35 (70)	2 (40)	37 (67)
8	7 (14)	1 (20)	8 (14)
9	2 (4)	1 (20)	3 (54)

### Preoperative SN mapping

Tracer preparation, injection and preoperative imaging (lymphoscintigraphy and SPECT/CT) were performed as previously described by KleinJan et al. [9].

Briefly, the hybrid tracer ^99m^Tc-nanocolloid was prepared by adding 2.0 mL pertechnetate (approximately 300 MBq) to a vial of nanocolloid (GE Healthcare, Eindhoven, The Netherlands). ICG (0.05 mL, 5.0 mg/mL: PULSION Medical, Feldkirchen, Germany) was then added to the vial of ^99m^Tc-nanocolloid. The total volume was subtracted from the volume of the vial and saline was added to reach a total volume of 2.0 mL in the syringe, after which the tracer was injected into the prostate under transrectal ultrasound guidance. The injection was followed by preoperative lymphoscintigraphy (15 min and 2 h after injection) and SPECT/CT imaging directly after acquisition of the late lymphoscintigram. After imaging acquisition the nuclear medicine physician determined the number and location of the SNs.

### Surgical (imaging) tools

For the surgical procedure a da Vinci Si system (Intuitive Surgical, Sunnyvale, CA, USA) with an integrated Firefly fluorescence laparoscope was used. Via the goggles of the master console, images obtained with the laparoscope are presented to the urologist. Using the controllers of the console the urologist is able to switch between the white light imaging mode and the fluorescence imaging mode of the fluorescence laparoscope. In the initial 50 patients fluorescence imaging with the standard settings was evaluated.

After a software upgrade (P9 software update) of the da Vinci Si system, the urologist was able to manually adjust the fluorescence illuminator settings. In this second set-up, with which 5 patients were evaluated, the intensity of the white light background in the fluorescence image was varied from 30 % (standard settings) to 15 % and 0 %. The visibility of the fluorescence signal within the SNs was evaluated at these three different settings.

### Surgical procedure

**Fig. 1:**
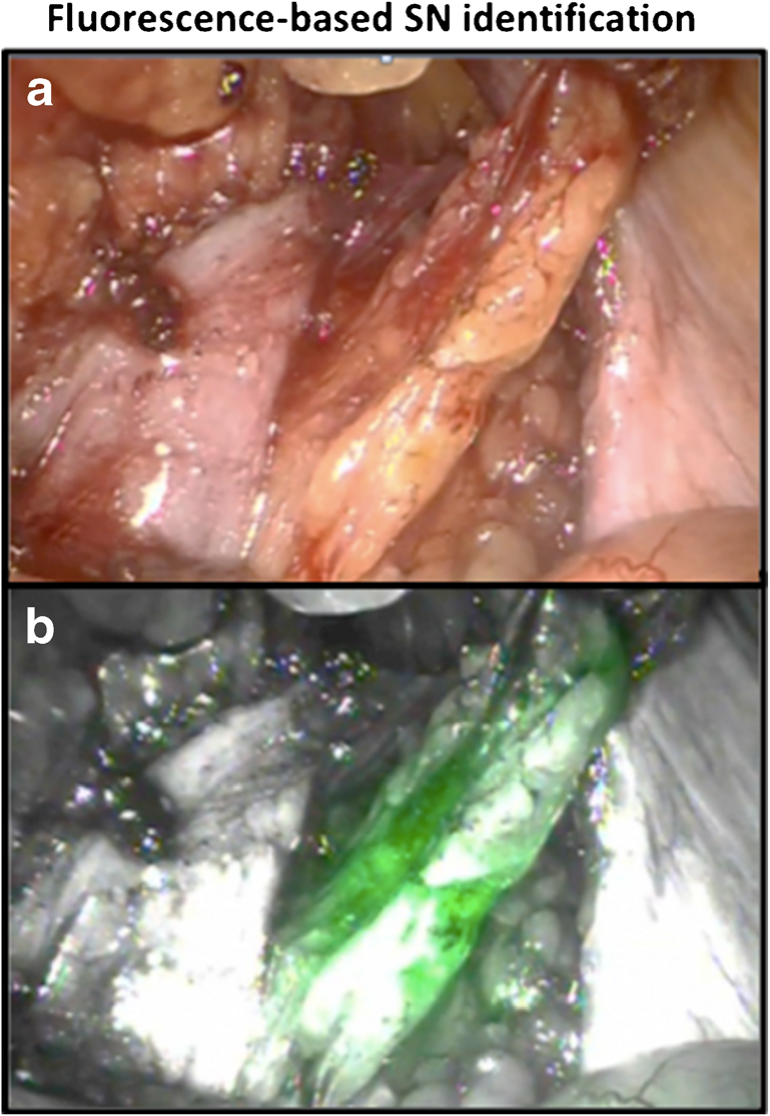
Fluorescence-based SN identification: **a** white light image; **b** fluorescence-based image

Prior to the start of the operation, the operating urologist viewed the SPECT/CT images using a DICOM viewer (PACS Vue Solutions; Carestream Health, Rochester, NY). The SNs identified on SPECT were again related to their anatomical context using the anatomical information provided by the low-dose CT scan. This information was used to help guide the positioning of the surgical tools and the fluorescence laparoscope for optimal SN localization and resection. Preopeatively identified SNs in a location with a high risk of surgical complications were left in situ. The remaining SNs, after initial preparation of the area of interest, were optically identified by switching between white light imaging and fluorescence imaging (Fig. 1). After excision of the SN, the surgical bed was re-examined using a combination of white light and fluorescence imaging.

To simplify the surgical workflow, based on the results of our previous study [9], no laparoscopic gamma probe was used intraoperatively. However, ex vivo the gamma probe (Europrobe 2; Eurorad S.A., Eckbolsheim, France) was used to validate the radioactive signature in the excised SNs/LNs. To confirm the presence of fluorescence in the excised SNs, fluorescence imaging was performed ex vivo using a fluorescence camera for open surgery (PDE; Hamamatsu Photonics K.K., Hamamatsu, Japan). The number and location of SNs identified preoperatively and intraoperatively were recorded together with their in vivo and ex vivo fluorescent and radioactive status.

Following SN excision, an ePLND was performed followed by the the radical prostatectomy. The ePLND included dissection of LNs from the following areas: around the external iliac artery and vein, within the obturator fossa, and surrounding the internal iliac artery [19].

### (Histo)pathological examination

(Histo)pathological analysis of the SNs, additional LNs from the ePLND specimens and the prostate were performed as previously described [9].

### Follow-up

Complications occurring within 90 days of the operation were recorded using the Clavien-Dindo classification and were evaluated in the initial 50 patients [20]. In the last 5 patients evaluated follow-up was not sufficiently long for them to be included in the postoperative evaluation. The findings were compared to those reported in our previous study in which we reported on fluorescence guidance without robotic integration [9].

### Statistical analysis

Statistical analysis was performed using SPSS Statistics, version 22 (SPSS Inc., Chicago, IL). A *p* - value <0.05 was considered significant. For continuous variables, the mean or median and interquartile ranges (IQR) are presented. For discrete variables, frequencies and percentages are reported. The Welch two-sample *t* - test was used to compare the preoperative Briganti scores, and the number of removed SNs and LNs in the patients in this study and in the patients in the previous study [9]. A Fisher’s exact test was used to compare the tumour positivity rate (pN0 or pN1), and to evaluate differences in postoperative complications between the initial 50 patients included in this study and the patients in the previous study [9].

The false-negative rate was calculated on a per-patient basis using the following formula: false-negative rate = [false-negative patients/(false-negative patients + true-positive patients)] × 100 %. The sensitivity of the SN biopsy procedure was also calculated on a per-patient basis using the following formula: [no. of true-positive patients/(no. of true-positive patients + no. of false-negative patients)] × 100 %.

## Results

### Preoperative imaging

**Fig. 2:**
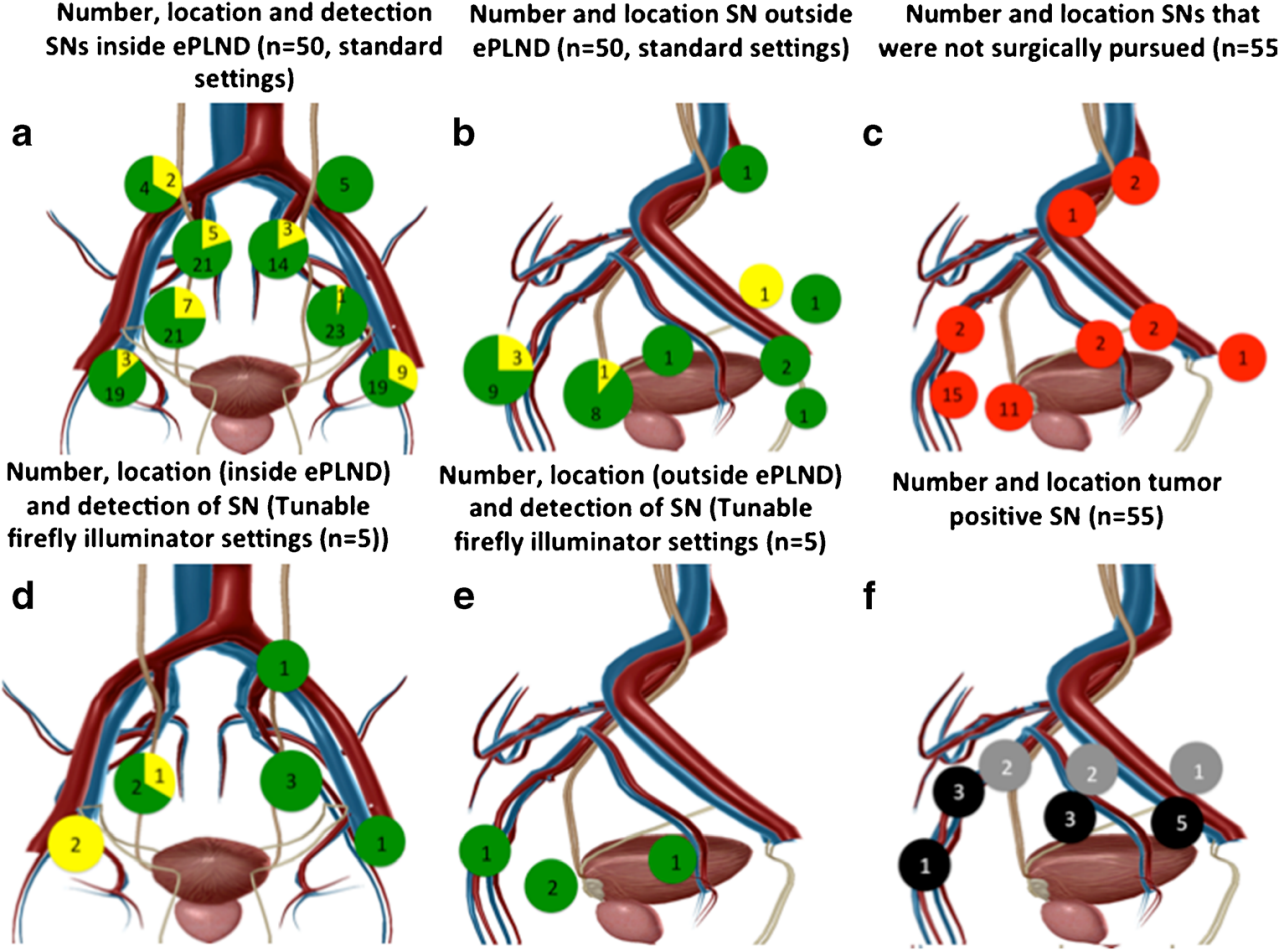
Locations of SNs and LNs detected intraoperatively. **a**, **b** SNs located inside and outside the ePLND area (*green* in vivo fluorescent SNs, *yellow* ​ex vivo identified SNs *n* = 50). **c** SNs that could not be removed (*red*, *n* = 55). **d**, **e** SNs located in the additionally included five patients inside and outside the ePLND area (*green* in vivo fluorescent SNs, *yellow* ​ex vivo identified SNs). **f** Location of tumour-positive SNs (*black* right-sided SNs, *grey* left-sided SNs). The images were generated using Visible Body software (Argosy Publishing, Newton Upper Falls, MA)

In 10 of the 55 patients (18.2 %) only unilateral drainage was observed. In the overall group of 55 patients, 147 SNs were identified on the lymphoscintigrams. An additional 65 SNs were identified on SPECT/CT imaging, resulting in a total of 212 SNs (median 4 per patient, IQR 3 – 5) identified on preoperative imaging. Of these 212 preoperatively identified SNs, 55 (26 %) were located outside the ePLND area. These results are specified for each patient group in Table 2 and in Fig. 2.

**Table 2:** SN detection and pathological evaluation

	Group		Total (*n* = 55)
	SN + ePLND (*n* = 50)	SN + ePLND, customized settings (*n* = 5)	
SNs detected preoperatively			
On lymphoscintigraphy, total	137	10	147
On lymphoscintigraphy, per patient, median (IQR)	2.5 (1 – 4)	2 (1 – 3)	2 (1 – 4)
On SPECT/CT, total	4 (3 – 5)	2 (1 – 3.5)	4 (3 – 5)
On SPECT/CT, per patient, median (IQR)	201	11	212
SNs detected intraoperatively			
Total removed	184	14	198
No. removed per patient, median (IQR)	4 (2 – 5)	1 (1 – 5)	4 (2 – 5)
No. not resected	36	0	36
No. additionally resected (as a result of SN cluster formation)	19	3	22
Pathological SN evaluation			
No. harvested per patient, median (IQR)	4 (2 – 6)	1 (1 – 5)	4 (2 – 5)
Total no.	226	30	256
Total no. tumour-positive	17	0	17
Pathological LN evaluation			
No. harvested from ePLND specimen per patient, median (range)	10 (8 – 14)	12 (7 – 22)	10 (8 – 15)
Total no.	582	69	651
Total no. tumour-positive (SNs + LNs)	41	0	41
Pathological SN + LN evaluation (total)			
Total no. removed per patient (SN + ePLND), median (IQR)	15.5 (12 – 20)	18 (12 – 28.5)	16 (12 – 20)
Total no. harvested SNs + LNs	807	99	906
Tumour-positive rate, no. (%) of patients pN1	1 4 (28)	0 (0)	14 (25)

### Intraoperative sentinel node identification

Of the 55 included patients, 54 were operated upon in a 1-day protocol with a median of 5.04 h between tracer injection and the start of the operation (IQR 4.33 – 5.20 h). One patient was operated 18.40 h after tracer injection. The preoperatively SPECT/CT images were analysed in the operating theatre using an interactive DICOM viewer and provided a virtual starting point for placement of the fluorescence laparoscope in localizing the SNs via fluorescence guidance.

Of the 201 SNs identified in the initial 50 patients on preoperative imaging, 36 (17.9 %) were not pursued during the operation. The decision not to resect these SNs was made on the basis of its anatomical location or relationship to close structures, such as presacral and pararectal SNs located close to the rectum or SNs located behind vascular structures such as the internal iliac artery, external iliac artery or vessels in the para-aortal region (Fig. 2c).

As a result of cluster formation, 19 extra SNs were surgically resected based on fluorescence identification. These clustered SNs were seen as one “hotspot” on SPECT, but the CT scan indicated multiple LNs at the location of the hotspot. A total of 148 SNs were removed using intraoperative fluorescence guidance (this was 80.4 % of the 184 surgically resected SNs; Table 2). After removal, ex vivo gamma tracing confirmed excision of these SNs.

The 19.6 % of SNs that could not be resected using fluorescence guidance were resected from the ePLND samples based on the anatomical information provided by the SPECT/CT images and ex vivo gamma tracing and fluorescence imaging. More detailed ex vivo analysis of the ePLND samples allowed fluorescence identification of 97.8 % (180) of the 184 SNs resected and detection of the radioactive signal in 100 % of the  SNs.

**Fig. 3:**
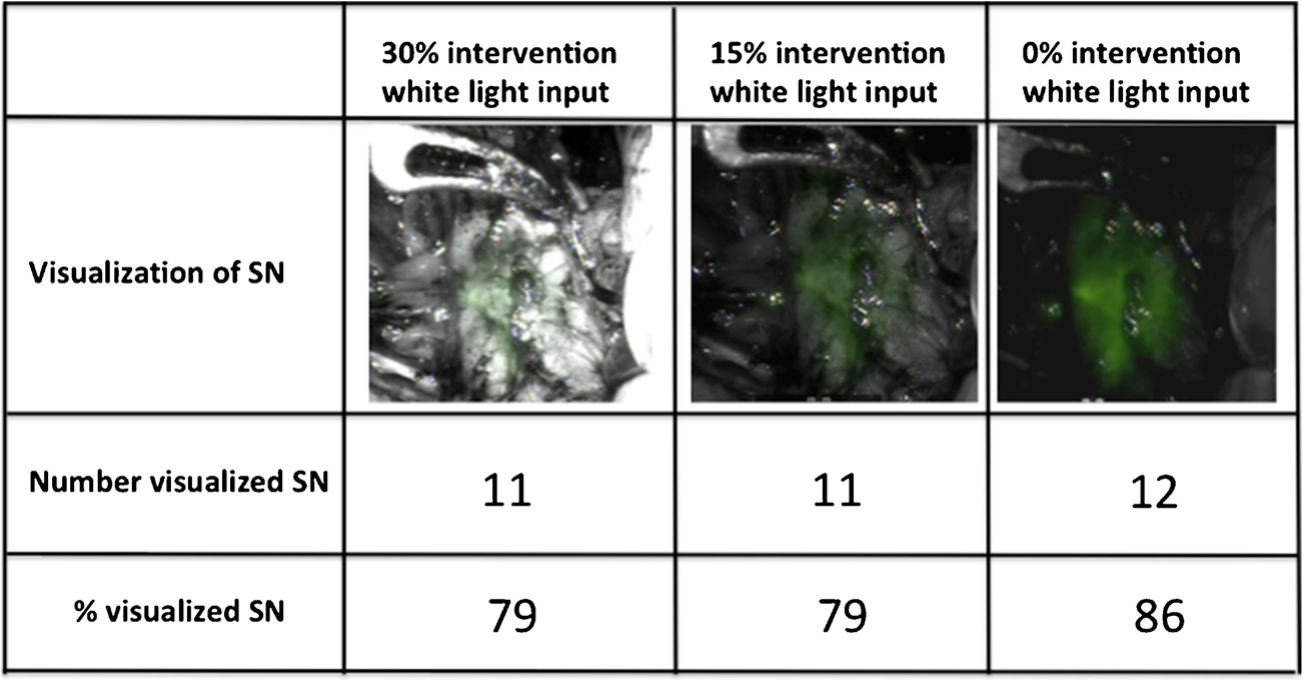
Evaluation of adjustable fluorescence settings. SNs identified with various fluorescence settings

In the five patients in whom the tunable fluorescence imaging settings were evaluated, as well as the 11 preoperatively identified SNs, 3 more SNs (part of a cluster of SNs) were removed under fluorescence guidance (Table 2). Of the 14 SNs, 11 (78.6 %), 11 (78.6 %) and 12 (85.7 %) were visualized at 30 %, 15 % and 0 % white light, respectively (ex vivo, 100 % of the SNs were fluorescent and radioactive). The effect of the light settings on the fluorescence image quality is shown in Fig. 3.

Because the fluorescence laparoscope is an integral part of the da Vinci Si system, the urologist manoeuvred and placed the fluorescence laparoscope independently of the surgical assistant, a feature that had a positive influence on the surgical logistics.

### Pathological examination SNs and ePLND specimens

At pathology, 256 nodes were extracted from the 198 SN specimens surgically excised from the 55 patients, (median 4 per patient, IQR 2 – 5). From the ePLND specimens a total of 651 LNs were removed, giving a median of 16 LNs per patient. The results are presented in more detail for each patient group in Table 2.

Histopathological examination revealed tumour-positive LNs in 25.4 % of patients (14 of 55 patients, total 41 tumour-positive nodes). In 10 of these 14 pN1 patients (71.4 %), the SNs were the only tumour-containing nodes. In 3 patients, tumour-positive LNs were also found next to the tumour-positive SNs. The result in one patient was false-negative. In this patient the SN was tumour-negative but a metastasis was detected in a LN from the ePLND tissue specimen, and this LN was not fluorescent or radioactive during surgery. The false-negative rate in this study was therefore 7.1 % (1/14). The sensitivity of SN biopsy in this cohort was good: the procedure correctly staged 13 of 14 node-positive patients (92.9 %). In a previously reported group of patients with similar Briganti scores, we found a tumour-positive node rate of 20 % (8/40 patients) [9]. Compared to the previous study, in the current study, significantly more LNs were removed per patient (median 16 vs. 12; *p* < 0.001) and 5.4 % more node-positive patients were identified (14/55 patients; i.e. 25.4 % vs. 20 %; *p* = 0.6). There was no significant difference in the a priori likelihood of LN metastasis (*p* = 0.3).

### Follow-up (complications)

No significant differences were found in the postoperative complication rates between the patients in the current study and those in the previous study (Table 3) [9]. Changing and integrating the fluorescence laparoscope did not change the postoperative complication rate.

**Table 3:** Complication rate

Complication	Clavien-Dindo grade	This study, group SN + ePLND (*n* = 50)	KleinJan et al. [9] (*n* = 40)	Total (n = 90)
Lymphocele	IIIa	1 (2)	2 (5)	3 (3)
Urinary tract infection	II	2 (4)	2 (5)	4 (4)
Postoperative bowel obstruction	II	1 (2)	1 (3)	2 (2)
Micturition obstruction (Sachse ureterotomy)	IIIb	0	1 (3)	1 (1)
Hematoma of the ventral abdominal wall	I	1 (2)	0	2 (9)
Postoperative infected abdominal haematoma	II	1 (2)	1 (3)	2 (9)
Leakage of anastomosis	I/III	2 (4)	0 (0)	2 (2)
Postoperative wound infection	II	0	1 (3)	1 (1)
Epididymitis	II	0	1 (3)	1 (1)
Hydronephrosis	IIIa	0	1 (3)	1 (1)
Deep venous thrombosis	II	1 (2)	0	1 (1)
Total		9 (18)	10 (25)*	19 (21)

Values are number (%) of patients

*p = 0.4

## Discussion

Integrating molecular imaging and robotic surgery is an important step in the evolution that is taking place in the treatment of prostate cancer. In the current study, using a hybrid (radioactive and fluorescent) SN tracer, findings from nuclear medicine imaging were used to guide the surgical resection using a robot-integrated fluorescence laparoscope. In some patients, it was decided, based on the SPECT/CT information, that some of the preoperatively identified SNs were not eligible for surgical removal. In the remaining patients, the anatomical locations derived from the SPECT/CT images were used to position the fluorescence laparoscope or, when intraoperative fluorescence imaging did not provide accurate SN identification, to guide resection of the SNs.

Robotic integration of fluorescence imaging gives the urologist more control over the use and positioning of the laparoscope, thus increasing his/her autonomy [21, 22]. To our surprise, using the standard fluorescence imaging settings of the robot-integrated fluorescence laparoscope, integration did not convert into an improved in vivo fluorescence-based SN visualization rate. In fact, the percentage found was lower than the rate we found previously in a setting where the bedside assistant was responsible for placement of an external fluorescence laparoscope [9]. This difference seems to be the result of differences in the camera technology used. However, it may also have been caused by the shorter time spent by the urologist in exploring the area of interest. The last point – saving surgical time to allow accurate fluorescence identification – seems to be in line with our previous finding [9]. Nevertheless, the fluorescence imaging miss-rate of 15 – 20 % (in the 55 patients) means that with fluorescence guidance alone a large number of SNs would have been missed. Due to the hybrid nature of the tracer used, the radioactive signature could be used to compensate for this shortcoming and to identify the additional SNs. Rather than using cumbersome laparoscopic gamma tracing, which again has to be performed by the bedside assistant, we used a more time effective combination of ex vivo gamma tracing and SPECT/CT images to guide resection of the residual SNs. Next to the radioactive signatures, the fluorescent signatures we verified ex vivo considerably increased the fluorescence-based SN detection rate (>97 %). This finding emphasizes that the tissue attenuation encountered in the in vivo situation limits the success of the fluorescence guidance approach, rather than the presence of tracer in the SNs. It also indicates that the backup from SPECT/CT is of the utmost importance. In an ongoing randomized controlled study we are evaluating these aspects in more detail.

In the five patients in whom the fluorescence settings could be adjusted, decreasing the percentage of white light, and thus decreasing the level of anatomical detail in the fluorescence image, helped improve the detection rate to 85.7 %. This improvement seems to contradict our previous findings. With a different fluorescence laparoscope, the introduction of white-light anatomical background information positively influenced the intraoperative fluorescence-based identification rate [9]. In this previous set-up we did not determine if the application of flexible white-light settings could improve fluorescence detection rates even further. The increased time used for the fluorescence detection at different light settings could also have played influenced the detection rate. Based on the current findings, an adjustable illuminator, wherein white light intensity can be tailored, may most flexibly accommodate the urologist’s needs.

One may reason that increasing the proportion of tumour-positive LNs resected will increase the likelihood of the patient’s recovery from the disease. In this sense, the 5.4 % greater rate of detection of tumour-positive LNs and the higher sensitivity than in our previous study (92.9 % vs. 75.0 %) [9] can be considered valuable. However, we cannot fully attribute this result to the surgical procedure. The tumour-positive LN detection rate may partly also be affected by the increased number of resected SNs and the pathological examination of the specimens. SNs have a higher chance of containing (micro)metastases and thus have a prognostic value [23] and as such receive more careful pathological evaluation including immunohistochemistry and cutting deeper levels than LNs out of a dissection template [24–27].

A crucial aspect of the clinical introduction of a new technology is the evaluation of the influence the technology will have on clinical outcome. For fluorescence guidance technologies such information is limited [14]. While one may reason that the SN biopsy procedure increases the chance of postoperative complications, the complication rate we observed was lower than in our previous study (*p* = 0.4) [9]. In both studies this result may in part be a consequence of using SPECT/CT planning to guide the procedure away from SNs located in areas that may be associated with postoperative complications.

The extensive introduction of new surgical technologies means that a need has been established for platforms that allow the integration of numerous imaging findings into the surgical workflow. In our view the da Vinci robot platform may act as such a linking technology. For example, robotic surgical goggles have been used to introduce complementary intraoperative and preoperative imaging findings as well as virtual/augmented reality displays [12, 28]. Since in the current study it was not possible to interactively examine the DICOM SPECT/CT images in Tilepro® during the operation, the urologist had to leave the console to examine them on a separate DICOM viewing station. However, in another study we have already provided a proof-of-concept that directly linking SPECT/CT imaging information and fluorescence imaging during robot-assisted procedures may provide the next step in surgical guidance [29].

The hybrid tracer concept illustrated in this study and our previous prostate cancer-related studies [9, 10, 30] provides a valuable extension of routine SN identification. A similar hybrid concept holds promise for (tumour-)targeted tracers and may benefit from the set up we have applied for SN identification. One example of a target that holds promise for image-guided resection is prostate-specific membrane antigen (PSMA), which is already routinely used in PET/CT-based diagnostics (^68^Ga-PSMA-HBED-CC) and has been successfully used for radioguided resection (^111^In-PSMA I&T) [31]. Theoretically, a hybrid PSMA derivative [32] would extend these efforts to accommodate (robot-integrated) hybrid surgical guidance towards the primary tumour margins, lymphatic metastases, and possibly even other distant metastases.

### Conclusion

Hybrid tracers help to integrate nuclear medicine and fluorescence-guided robotic surgery, but the use of a robot-integrated fluorescence laparoscope did not improve fluorescence-based SN identification. Hence, 3D preoperative imaging information from nuclear medicine remains crucial for (virtual) planning of complex surgical resections of multifocal lesions. Further technical refinement of robot-integrated guidance modalities in surgical procedures should improve the relationship between preoperative and intraoperative imaging findings.
